# 

**Published:** 1892-10-22

**Authors:** 


**Notices of Births, Deaths, and Marriages,** are inserted in this column at a charge of 2s. 6d. for an announcement not exceeding four lines.

### NOTICE TO CORRESPONDENTS.

All MS., Letters, Books for Review, and other matters intended for the Editor should be addressed The Editor, The Lodge, Porchester Square, London, W.

The Editor cannot undertake to return rejected M.S., even when accompanied by stamped directed envelope.

**Notice to Advertisers and Subscribers.**

All Advertisements, Orders for Copies of The Hospital, and Business Communications should be addressed to The Publisher, *not to the Editor*, The Hospital, 140, Strand, W.C.

The Cost of Subscription, post free, is as follows:—Per week, 2½d.; per quarter, 2s. 6d.; per half-year, 5s.; per annum, 8s. 6d. Foreign:—Per annum, 10s. 8d.; payable, in all cases, in advance.

**"Burdett's Hospital Annual," 1891—1892.**

Third year of publication. 600 pp. Boards, gilt lettering. A most complete guide to British and Colonial Hospitals, Dispensaries, Nursing and Convalescent Institutions. and Asylums. Price 3s. 6d. Published by The Scientific Press (Limited), 140, Strand, W.C.

**NOTICE.—The Index for Vol. XI. (ending March, 1892) is on sale at the Office of this Journal, price 2½d., post free.**

### Books Received.

The Scientific Press.

"The Art of Massage." By Mrs. Creighton Hale.

Fisher Unwin.
"Household Nursing." By John Ogle Tunstall, M.D.

Lewis and Co.

"Medical Microscopy." By Frank Wethered, M.D.

**PAMPHLETS.**

Adlard and Son.
"Four Hundred Cases of Phthisis." By F. Sandwith, M.D.
"Report of the Health and Sanitary Condition of the County of Lanark for 1891."

### The Student of Medicine.

[All communications for this Supplement should be addressed to The Editor of The Hospital, at 140, Strand, with the words, "Students' Column," written in the left hand top corner of the envelope.]

### NOTES FROM THE SCHOOL.

**Charing Cross Hospital Medical School.**

The scholarship of 60 guineas, open to students of the universities of Oxford and Cambridge, has been awarded to Mr. Walter S. Sheppard, of Christ's College, Cambridge. The entrance scholarship of 120 guineas has been awarded to Mr. Sidney Hubert Berry, and that of 60 guineas to Mr. Alfred Burn.

**University College, London.**

Her Majesty's Commissioners for the exhibition of 1851 have placed at the disposal of the council of the college the nomination to a scholarship of £150 for the year 1893. The scholarship is intended "to enable students who have passed through a college curriculum to continue the prosecution of science with the view of aiding in its advance, or in its application to the industries of the country." Out of the late Mr. Berridge's Charity Legacy, the trustees have allotted to University College the sum of £10,000, for the promotion of the study of hygiene. The following gentlemen have been recommended for medical entrance exhibitions: Mr. C. Bolton, £100; Mr. W. R. Battye, £60; Mr. E. P. Chennells, £40.

### APPOINTMENTS.

Brockbank, E. M., M.B., appointed House Physician at Birmingham General Hospital. Brush, G. C., M.B., C.M.Edin., appointed Clinical Assistant to the Shoreditch Infirmary. Bryce, Thomas H., M.A., M.B., F.F.P.S.G., appointed Extra-Dispensary Surgeon to the Western Infirmary, Glasgow. Campbell, W. S., M.B., C.M.Glas., appointed Medical Officer for the Crediton District and Workhouse of the Crediton Union. Cluckie, N. G., M.B., L.F.P.S.Glas., appointed Oculist to the New Public Eye Dispensary, Dumbarton. Cross, A. J., M.B., C.M.Edin., appointed Medical Officer for the Dalton District of the Ulverston Union. Dickson, T. H., M.B., B.C., L.R.C.P., appointed Medical Inspector of Her Majesty's Customs. Evers, Charles John, M.B.Durh., M.R.C.S.Eng., appointed Medical Officer for the Faversham Port District. Foot, Ernest G., M.R.C.S.Eng., L.S.A., appointed Medical Officer for No. 4 District of the Petworth Union. Foxcroft, F. W., M.B., C.M., appointed House Physician at Birmingham General Hospital. Frier, Charles, M.B.. C.M.Edin., appointed House Surgeon to the Royal Surrey County Hospital, Guildford. Green, Reginald, M.B., B.S.Durh., appointed Assistant Medical to the Gateshead Dispensary. Gulland, G. Lovell, M.A., M.D., F.R.C.P.Edin., appointed Assistant-Physician to the Edinburgh Royal Infirmary. Heelas, Walter W., M.R.C.S., L.R.C.P. Lond., appointed House Physician to the General Lying-in Hospital, York Road, Lambeth, S.E. Hooper, Alfred, M.R.C.S. Eng., L.S.A., re-appointed Medical Officer of Health to the Burton-on-Trent Rural Sanitary Authority. Hyatt, J. T., L.R.C.P.Edin., M.R.C.S.Eng., appointed District Medical Officer of the Shepton Mallet Union. Irwin, Mr. S., appointed Medical Officer for the Almondsbury District of the Thornbury Union. James, Alexander, M.D., F.R.C.P.Edin., appointed Ordinary Physician to the Edinburgh Royal Infirmary. James, W. E., M.R.C.S., L.R.C.P.Lond., appointed Medical Officer of Health for the Abercarn Urban Sanitary District of the Newport (Mon.) Union. Kempe, Arthur Wightman, M.D., M.R.C.S.Eng., re-appointed Medical Officer of Health to the Exmouth Local Board. Lancaster, Ernest Le Cronier, B.A., M.B., B.Ch.Oxon., M.R.C.S.Eng., appointed Chloroformist and Pathologist to the Swansea Hospital. Larking, A. E., M.D.Durh., M.R.C.S.Eng., appointed Medical Officer for the Eighth District of the Aylesbury Union. MacGillivray, C. Watson, M.D., F.R.C.S.Edin., appointed Ordinary Surgeon to the Edinburgh Royal Infirmary. Macnaughton, G. W. F., M.S.Edin., M.B.Edin., appointed Third Assistant Medical Officer at the Worcester County and City Lunatic Asylum. Marriott, Hyde, M.B., B.Sc.Lond., appointed Honorary Surgeon to the Stockport Infirmary. Napier, James, appointed Public Analyst for the County of West Suffolk. Nicoll, Jas. H., M.B., C.M.Glasg., Dispensary Surgeon Glasgow Western Infirmary, appointed Surgeon to the Depart'

THE STUDENT OF MEDICINE—(*continued*).

ment for Diseases of the Urinary and Genito-urinary Organs in the Glasgow Central (late Anderson's College) Dispensary. Paine, W. H., L.R.C.P.Lond., M.R.C.S.Eng., appointed Medical Officer for the West Green District of the Edmonton Union. Parkinson, C. H. W., M.R.C.S., D.P.H.R.C.P.Edin., appointed Medical Officer of Health to the Wimborne Local Board. Pearce, J. P., M.R.C.S., appointed Medical Officer of Health for the Lewes Urban Sanitary District of the Lewes Union. Stanfield, Wm., M.D.St.And., L.R.C.P.Edin., M.R.C.S., re-appointed Medical Officer of Health for Lees, Manchester. Stephens, William John, L.R.C.P.Edin., L.S.A., appointed Surgeon to the Brighton Borough Police Force. Stockman, Ralph, M.D., F.R.C.P.Edin., F.R.S.E., appointed Assistant Physician to the Edinburgh Royal Infirmary. Stockwell, F. B., M.D., M.R.C.S. Eng., appointed District Medical Officer of the Shepton Mallet Union. Thompson, Alexis, M.D., F.R.C.S.Edin., appointed Assistant-Surgeon to the Edinburgh Royal Infirmary. Tweedy, R. C., M.R.C.S., L.R.C.P., appointed Assistant House-Surgeon at Birmingham General Hospital. Vinrace, Felix, M.D., F.R.C.S., appointed Surgical Casualty Officer at Birmingham General Hospital. Wallace, David, M.D.Edin., M.R.C.S.Eng., appointed Assistant-Surgeon to the Edinburgh Royal Infirmary. Ware, H. S., B.A., M.B., B.C.Camb., L.S.A., appointed House-Physician to the City of London Hospital for Diseases of the Chest, Victoria Park, E. Yarrow, Geo. Eugene, M.D., L.R.C.P. Lond., re-appointed Surgeon to the G (or Old Street) Division of the Metropolitan Police.

### VACANCIES.

The following vacancies are announced this week, the amount of salary in each case being given after the name of the institution:

### Resident Medical Officers.

Birmingham General Hospital, Resident Surgeon, doubly qualified.
Borough of Kingston-upon-Thames, Medical Officer of Health — Salary £150 per annum.
Devon County Lunatic Asylum, Assistant Medical Officer; unmarried—Salary £100 per annum, rising to £120, with board, lodging, and washing.
Manchester Clinical Hospital for Women and Children, Park Place, Cheetham Hill Road, Manchester, House Surgeon—Salary £80 per annum, with apartments and board.
Rotherham Hospital and Dispensary.—Assistant House Surgeon—Rooms, commons, and washing provided.
Royal Free Hospital, Gray's Inn Road, W.C., Junior Resident Medical Officer, doubly qualified—Board and residence provided.
Weston-Super-Mare Hospital and Dispensary, Medical Officer to the Provident Dispensary attached to the Hospital; doubly qualified—Salary £60 per annum with board, lodging, and washing.

### Non-Resident Medical Officers.

Birmingham General Hospital, Pathologist—Salary £120 per annum.
Sheffield School of Medicine, Tutor, to take charge of dissecting rooms and hold classes in Anatomy and Physiology—Salary commencing £100 per annum.
Huddersfield Infirmary, Honorary Physician.
Leeds Friendly Societies' Medical Association.—Assistant Medical Officer—Salary to commence £120 per annum and midwifery fees.
North Dublin Union, Finglas and Glasnevin Dispensary, Medical Officer—Salary £170 per annum and fees.

